# 30-minute CMR for common clinical indications: a Society for Cardiovascular Magnetic Resonance white paper

**DOI:** 10.1186/s12968-022-00844-6

**Published:** 2022-03-01

**Authors:** Subha V. Raman, Michael Markl, Amit R. Patel, Jennifer Bryant, Bradley D. Allen, Sven Plein, Nicole Seiberlich

**Affiliations:** 1 Division of Cardiovascular Medicine and Krannert CV Research Center, Indiana University School of Medicine, Indianapolis, IN USA; 2 Department of Radiology, Feinberg School of Medicine, Northwestern University, Chicago, IL USA; 3 Section of Cardiology, Department of Medicine, University of Chicago, Chicago, IL USA; 4 Department of Cardiology, National Heart Centre Singapore, Singapore, Singapore; 5 Multidisciplinary Cardiovascular Research Centre and Biomedical Imaging Science Department, Leeds Institute of Cardiovascular and Metabolic Medicine, University of Leeds, Leeds, UK; 6 Department of Radiology, University of Michigan, 1150 West Medical Center Drive, Ann Arbor, MI 48109 USA; 7 Cardiovascular Institute, IU Health, Indianapolis, IN USA; 8 Department of Biomedical Engineering, McCormick School of Engineering, Northwestern University, Evanston, IL USA

**Keywords:** Cardiovascular magnetic resonance, Magnetic resonance imaging, Cardiomyopathy, Ventricular arrhythmia, Ischemic heart disease, Myocarditis, Clinical practice

## Abstract

**Background:**

Despite decades of accruing evidence supporting the clinical utility of cardiovascular magnetic resonance (CMR), adoption of CMR in routine cardiovascular practice remains limited in many regions of the world. Persistent use of long scan times of 60 min or more contributes to limited adoption, though techniques available on most scanners afford routine CMR examination within 30 min. Incorporating such techniques into standardize protocols can answer common clinical questions in daily practice, including those related to heart failure, cardiomyopathy, ventricular arrhythmia, ischemic heart disease, and non-ischemic myocardial injury.

**Body:**

In this white paper, we describe CMR protocols of 30 min or shorter duration with routine techniques with or without stress perfusion, plus specific approaches in patient and scanner room preparation for efficiency. Minimum requirements for the scanner gradient system, coil hardware and pulse sequences are detailed. Recent advances such as quantitative myocardial mapping and other add-on acquisitions can be incorporated into the proposed protocols without significant extension of scan duration for most patients.

**Conclusion:**

Common questions in clinical cardiovascular practice can be answered in routine CMR protocols under 30 min; their incorporation warrants consideration to facilitate increased access to CMR worldwide.

## Background

Over several decades, considerable evidence has accrued supporting cardiovascular magnetic resonance (CMR) as the standard for evaluating many common conditions [1], and this evidence is increasingly reflected in cardiovascular care guidelines [2–14]. Healthcare practices and facilities seeking to adhere to contemporary evidence and guidelines by providing CMR for their patient populations recognize this modality’s many advantages over other diagnostic approaches. These include lack of ionizing radiation needed with computed tomography (CT), single photon emission computed tomography (SPECT), and positron emission tomography (PET); diagnostic data impervious to body habitus issues that limit acoustic windows with echocardiography [15]; sampling of the entire myocardium without risk posed by invasive tissue procurement; detailed characterization with more accurate evaluation of 3-dimensional anatomy [16]; and greater value when considering cost and outcomes across a broad range of settings and conditions [17–19]. In particular, the unique sensitivity of CMR to subtle changes in heart muscle tissue structure and function make it indispensable when faced with many common questions that impact management in cardiovascular practice, including: (1) what is the etiology of heart failure or cardiomyopathy? (2) what is the substrate for ventricular arrhythmia? (3) does the patient have ischemic and viable myocardium? and (4) is there inflammatory myocardial injury? [20].

Recognition of CMR’s value in daily practice has improved as more expert radiologists and cardiologists are trained to perform and interpret CMR, and referring clinicians receive education on evidence supporting CMR’s utility. Patients expect value for the time and expense they incur for diagnostic testing, as well as better outcomes thanks to effective management guided by accurate diagnosis.

Despite being around for decades with a robust base of evidence supporting its utility in clinical practice, CMR remains poorly accessible to too many patients. This is especially problematic in community settings where magnetic resonance (MR) scanners are heavily scheduled for the full range of non-cardiac indications. Historic time slots of 60 min or longer hinder improved access to CMR in these and other settings. Time pressures incurred by longer exam times impact not just patients but also facilities and providers, as current reimbursement rates in many parts of the world may make growth in access to CMR financially difficult. In some respects, the vast armamentarium of CMR techniques developed over 30+ years of innovation has become a barrier to implementing CMR in routine clinical practice if they are routinely added to clinical protocols without due regard to scan duration adequate to address the clinical questions. Importantly, many of the most common clinical questions in cardiovascular practice can be addressed with rapid, core protocols that can be implemented on most current MR scanners [21, 22].

In this white paper, fully aligned with international cardiovascular practice guidelines and Society for Cardiovascular Magnetic Resonance standards for high quality CMR [23], we offer a basic 30-min or shorter CMR exam that answers many of the common clinical questions in cardiovascular practice including those articulated above.

### Efficient CMR for common clinical questions

Table 1 summarizes 4 common clinical questions answerable by a standardized 30-min CMR exam. In addition to these questions, the proposed protocols can be used in many other clinical scenarios as they form the core for every CMR exam.

**Table 1:** Common clinical questions answerable by a standardized 30-minute CMR exam

Common clinical questions	Extended questions answered CMR
Why is there heart failure or cardiomyopathy?	What are the left and right ventricular volumes and ejection fractions?^†^ Is there infarct scar, or is it non-ischemic disease? What type of any infiltrate is present (e.g., sarcoid granuloma, amyloid protein, sphingolipid, iron)?
What is the substrate for ventricular arrhythmia?	What type of any myocardial or structural heart disease is present?
What is the extent of ischemic and viable myocardium?	How much myocardium is infarcted? How much myocardium is ischemic? Is there concomitant non-ischemic myocardial disease? Is there significant mitral regurgitation?*
Is there ischemic or non-ischemic injury?	Is the troponin elevation due to inflammation or ischemic injury?

^†^Answered for all common clinical questions

*Add velocity-encoded cine to measure aortic and pulmonary artery stroke volume

#### Heart failure or cardiomyopathy

Precision in diagnosis is essential to guide effective treatment for patients with heart failure or cardiomyopathy [10, 24]. Common clinical questions include: What are the left ventricular (LV) ejection fraction (LVEF) and right ventricular (RV) ejection fractions (RVEF)? What is the underlying cause of ventricular dysfunction? Does this patient need an implantable cardioverter-defibrillator (ICD)? Should family members be screened?

While serological assays can complement imaging in the evaluation of metabolic and other uncommon non-myocardial causes of heart failure, direct evaluation of the myocardium with non-invasive imaging is usually required to make the diagnosis that guides effective management.

Well-established CMR techniques characterize the myocardium and define ventricular size, morphology, and function. Cine imaging with CMR is the reference standard for calculation of both LVEF and RVEF and allows accurate classification of heart failure with preserved ejection fraction (HFpEF), heart failure with mid-range ejection fraction (HFmEF), or heart failure with reduced ejection fraction (HFrEF) with treatment consequences [16, 25]. CMR yields excellent delineation of cardiac morphology to identify dilated [26], hypertrophic [26] and noncompacted myocardium [27, 28] and combinations of these findings with implications not just for individual patients but also for family members at risk of heritable conditions [29–31].

Myocardial disease resulting in heart failure or cardiomyopathy may span ischemic injury, non-ischemic fibrosis, infiltrative processes, or inflammatory damage. The workhorse CMR technique of late gadolinium enhancement imaging (LGE) can help distinguish all of these by the pattern of myocardial enhancement—subendocardial indicating infarct scar from ischemic injury, midwall typically predominant in fibrosis of non-ischemic cardiomyopathy, and epicardial typically predominant in inflammatory damage [32, 33]. Further, LGE can indicate the presence of infiltrative disorders such as sarcoidosis [34, 35] and amyloidosis [36]. Importantly, these LGE patterns have all been corroborated by histopathology [37].

#### Ventricular arrhythmia substrate

Why does my patient have ventricular arrhythmia? The answer to this question is critical, as ventricular arrhythmias can be life-threatening and often denote the presence of underlying heart disease. An understanding of the mechanism of the ventricular arrhythmia can help define prognosis and guide treatment. Ventricular arrhythmias are often mediated by reentry around or triggered activity from within damaged myocardium. Because of its ability to accurately characterize cardiac structure and function, CMR is well suited to diagnose a wide range of underlying cardiomyopathies known to be associated with ventricular arrhythmias [38–40].

Some patients with known cardiomyopathy are at increased risk of sudden cardiac death due to the occurrence of ventricular arrhythmias. Currently, LVEF is one of the major parameters used to identify patients most likely to benefit from primary prevention ICD placement [41]. However, patients with reduced LVEF may never experience a life-threatening ventricular arrhythmia, and other patients suffer sudden cardiac death despite having preserved LVEF. Pontone et al*.* have shown that CMR-based LVEF combined with myocardial characterization with LGE is superior to echocardiography for identifying individuals who will have ventricular arrhythmias benefiting from ICD placement [42]. The value of LGE as a biomarker for arrhythmic risk has been shown across ischemic heart disease, dilated cardiomyopathy, hypertrophic cardiomyopathy, and infiltrative cardiomyopathies [43–45].

#### Ischemic heart disease

Some of the main questions in the management of patients with new or known ischemic heart disease (IHD) include: Is there scar from previous myocardial infarction? Are my patient’s exertional symptoms the result of myocardial ischemia and, if so, what is the extent of ischemic and viable myocardium? If there is coronary artery disease (CAD) and ventricular dysfunction or heart failure, will ventricular function improve with revascularization? Is there concomitant non-ischemic disease explaining dysfunction beyond the severity of CAD?

CMR LGE imaging is the reference standard for visualization of infarct scar [46]. The absence of myocardial scar is not only indicative of myocardial viability but also associates with better patient prognosis. Indeed, even thinned and dyskinetic myocardial segments supplied by flow-limiting coronary stenosis can recover function following revascularization if LGE demonstrates less than 50% transmural extent of scar [47, 48].

Stress CMR with perfusion imaging combined with a pharmacologic agent, commonly a vasodilating drug such as adenosine or regadenoson, is superior to conventional stress imaging modalities such as SPECT and echocardiography to define the presence and extent of ischemic myocardium [49–51]. Indeed, vasodilator stress CMR is equivalent in terms of patient outcomes—and at lower cost—compared to the invasive reference standard measurement of coronary fractional flow reserve (FFR) [52]. Large registries have additionally shown that patients with a normal stress CMR exam have an excellent prognosis, whereas those with an abnormal stress CMR exam have an increased rate of major adverse cardiac events [53–55]. As such, guidelines on chest pain evaluation recognize a number of appropriate indications for stress CMR, for both those with as well as those without known coronary heart disease [56].

#### Myocardial inflammation

Clinicians often see patients who present with chest pain with no evidence of significant CAD, including those with myocardial infarction with no obstructive coronary arteries (MINOCA). Common questions in these patients include: Why does my patient have acute symptoms, abnormal cardiac biomarkers but no epicardial coronary artery disease? The differential diagnosis includes plaque rupture, embolism, coronary spasm, and microvascular causes. Another major cause includes non-ischemic myocardial inflammation or myocarditis, a mechanism highlighted by the severe acute respiratory syndrome coronavirus-2 (SARS-CoV-2) pandemic. Here, CMR again is the standard for appropriate diagnosis and management and the only non-invasive test that can accurately differentiate between ischemic and non-ischemic cardiac injury while also detecting myocardial and pericardial inflammation [57].

In all of the above scenarios, rapid CMR protocols of less than 30-min duration can provide essential information that guides patient management.

### Requisite CMR infrastructure

#### Workflow

Delivering an effective and rapid CMR service extends beyond the use of rapid imaging protocols. A well-coordinated team of administrative staff, clinicians and technologists is essential for efficient workflow.

*The team:* While there are many CMR team models around the world, most successful ones include one or more CMR physician champions. These are typically cardiologists or radiologists who have undergone training in CMR, and who are dependable and responsive leaders with an interest in managing CMR operations and personnel. Ideally, a consistent group of technologists performs all the CMR exams at a given facility to ensure consistency and to maintain efficiency in practice. The team is rounded out by a nurse or someone suitably qualified in vasodilator drug administration and appropriate clinical monitoring during stress exams. Each member of the CMR team can benefit from the relative efficiency and simplicity of the implemented rapid CMR protocol.

*The referral:* Educating referring physicians is important to ensure appropriateness and completeness of referrals. When suitable clinical detail is obtained, the CMR referral can be classed as suitable for rapid scan or if it requires a lengthier protocol such as evaluations for congenital heart disease or cardiac masses.

*Patient preparation:* Efficient patient preparation and screening of patients outside the scanner area can improve workflows by ensuring that the ‘next patient’ is getting ready while the ‘current patient’ is being scanned. Preparation includes screening for CMR contraindications, placement of an intravenous catheter for contrast injection, and placement of CMR-compatible electrocardiographic (ECG) electrodes. Other preparatory steps that can be taken outside the CMR scanner room are listed in Table 2. While outside the CMR room, the patient should also be educated about what to expect and have an opportunity to practice breath-holds, etc. which will help make image acquisition more efficient.

**Table 2:** Preparation for a CMR scan

Patient preparation
Brief medical history and screening for CMR contra-indications
Measurement of body height and weight
Explain the scan and what to expect to the patient
Practice breath-holding
Prepare the skin and place ECG electrode patches
Place intravenous catheters (1 for non-stress and 1–2 for stress scans, depending on the stress agent)
Clean scanner and room
Prepare the power injector
Prepare the ECG leads and coils

*ECG* electrocardiogram

*Preparation of the CMR scanner room:* In addition to preparing the patient, the CMR room should also be prepared. As an example, the power injector and other equipment could be prepared by the additional staff while the primary staff is positioning the patient on the CMR table. Having all the equipment prepared prior to starting the scan, with intravenous equipment positioned at the front of the scanner, will prevent time-consuming interruptions that require pulling the table out and entering the room during the middle of the scan. ECG gating should be optimized as required if initially sup-optimal with lead relocation and improving skin contact. Timely switching to peripheral pulse gating may be warranted to maintain rapid scanning.

#### Hardware, scanner platform, and safety for rapid CMR

*Field strength:* Performing CMR requires several pieces of hardware, starting with the CMR scanner. Scanner field strengths commonly available across clinical practices include 1.5 T and 3 T; rapid CMR can be done on both [58]. Advantages of 3T include inherently higher signal-to-noise ratio (SNR) that can result in improved image quality for CMR techniques such as first pass perfusion and LGE. Alternatively, the increase in SNR at 3T can be exploited for higher imaging acceleration factors and shorter scan time. However, in clinical practice, 1.5T systems are often preferred due to more reliable cardiac gating, lower specific absorption rate (SAR), and reduced image artifacts—especially for cine imaging [58].

*Gradient performance and coil systems:* All 1.5T and 3T CMR scanners manufactured after 2005 by the most prevalent CMR vendors (Siemens Healthineers, General Electric Healthcare, Philips Healthcare, United Imaging, Canon) can deliver the 30-min CMR protocol with high quality. Even many older platforms have maximum gradient amplitudes above 33 mT/m and slew rates above 120 mT/m/ms, enabling the rapid signal encoding and data collection required in CMR. For 3 T systems, higher gradient performance is recommended to reduced sensitivity to artifacts for cine imaging. These scanners are also typically equipped with receiver coil arrays with at least 8 independent channels, making parallel imaging with acceleration factors of R = 2 or 3 possible. Moreover, these systems can be supplied with the “cardiac package” by the manufacturer, which include (at a minimum) the scans needed to conduct a successful and high-quality exam as described in more detail below. Table 2 details minimum and optimal gradient systems, receiver coil, and sequences for CMR (Table 3).

**Table 3:** Minimum and Optimal Hardware and Pulse Sequences for CMR

Gradient system		Chest receive coil		Pulse sequences	
Minimum	Optimal	Minimum	Optimal	Minimum	Optimal
Amplitudes > 33 mT/m Slew rates > 120 mT/m/ms	Amplitudes ≥ 40 mT/m Slew rates ≥ 200 mT/m/ms	8	16 or higher	Localizer Cine Imaging Perfusion (for stress) LGE	Minimum plus - T1 and T2 mapping Velocity-encoded cine

*Other hardware:* In addition to the CMR scanner itself, CMR-specific equipment that can interface with the scanner are needed. ECG-gating hardware and software are required at the scanner to synchronize data collection with the patient’s heart rhythm. The software should be capable of prospective ECG triggering or retrospective ECG gating. A power injector capable of injecting gadolinium-based contrast agent, followed by saline flush at rates of at least 3 mL/s is needed for first-pass perfusion scans with an automated interface to ensure proper contrast injection timing.

*Safety:* The patient should be continuously monitored during the scan, especially the stress perfusion portion, using a CMR-compatible blood pressure cuff, the (scanner-generated) ECG and an in-scanner communication device. It is important to make sure that resuscitative equipment is maintained outside but close to the scanner room, including a defibrillator and appropriate emergency medications such as nitroglycerin, aminophylline, bronchodilators, epinephrine, atropine, antihistamines, intravenous fluid and oxygen. Although serious complications during vasodilator stress CMR are rare, sites should be prepared for them and may consider periodic “mock codes” as a team to practice the rapid removal of the patient from the scanner bore for resuscitation outside the scanner room. This further underscores the value of keeping CMR protocols short and focused on answering the clinical questions.

### Components of a standardized 30 min CMR exam

**Fig. 1:**
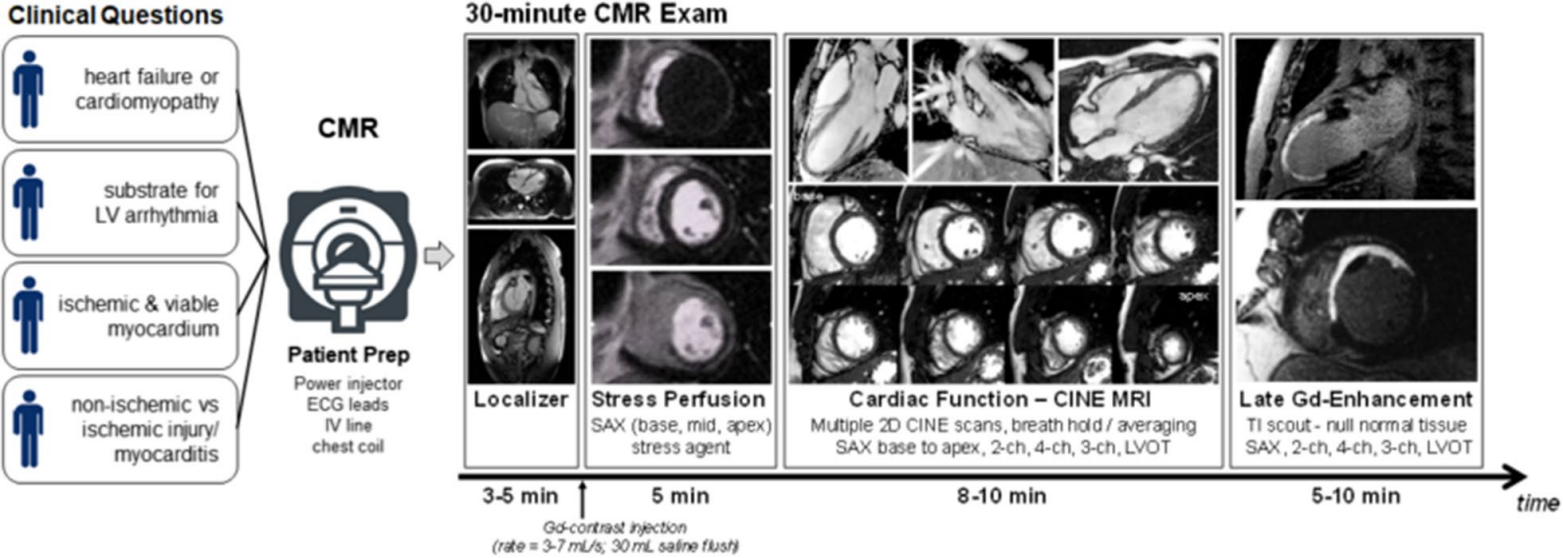
Typical Clinical Questions and Workflow for a 30-min CMR Exam: Many common questions in cardiovascular practice can be answered with cine, perfusion, and late gadolinium enhancement (LGE) imaging. Myocardial mapping, phase contrast imaging, and other sequences can easily be added to this workflow if available and useful to answer clinical questions for an individual patient

Well-established CMR techniques for localization, perfusion imaging, cine imaging, and LGE imaging available as part of any standard cardiac package can be combined into an efficient and highly standardized protocol for high quality CMR in under 30 min (Table 4, Fig. 1). Each of these is described in more detail below; together, these provide the essential information to answer the clinical questions summarized in Table 1 and described above. The reader is referred to an excellent series of ‘How I Do CMR’ publications and presentations on the Society for Cardiovascular Magnetic Resonance website (https://scmr.org/general/custom.asp?page=HowIDo) for more detail regarding prescription of cardiac planes, contrast dosing, and other practical tips.

**Table 4:** Components of a Standardized 30-min CMR Protocol

CMR technique	CMR sequence	Scan prescription	Scan time
Localizer	Single shot FSE, GRE, *or* bight blood bSSFP	3 orthogonal slices (axial, coronal, sagittal) Transverse stack covering the heart Standard cardiac planes: 2-chamber, 4-chamber, 3-chamber, short axis stack	5 min
Perfusion	Saturation recovery bSSFP *or* GRE	Short axis planes (base, mid, apex); Long-axis plane of choice Simultaneous intravenous injection of contrast (rate = 3–7 mL/s; 30 mL saline flush)	5 min
Cine Imaging	k-space segmented cine bSSFP *or* k-space segmented cine GRE e.g., to reduce susceptibility artifact	Standard cardiac planes: 2-chamber, 4-chamber, 3-chamber, LVOT, and shot axis stack (2D slices covering the heart from base to apex) Breath hold at end-expiration	8–10 min
LGE	2D inversion recovery (IR) bSSFP or GRE; optimal to include phase-sensitive inversion recovery reconstructions	Select inversion time that nulls normal myocardium, optimally with a mid-short axis plane TI scout acquisition Standard cardiac planes (same as cine imaging)	5–10 min

*bSSFP* balanced steady state free precession, *FSE* fast spin echo, *GRE* gradient echo, *IR* inversion recovery, *LVOT* left ventricular outflow tract

#### Localizers

Localizers are the initial images collected to assist in planning all other images. Because cardiac imaging planes are typically oblique in orientation, localizers are essential to ensuring that the subsequent scans depict the required cardiac anatomy and physiology in anatomically appropriate orientations.

Initial localizers span the standard 3 orthogonal slices (axial, coronal, sagittal), and are typically collected as single heartbeat acquisitions together in one short breath hold. Then, a stack of axial, single shot fast spin echo, gradient echo or bright blood balanced steady state free precession (bSSFP) images (8–10 mm slice thickness) is collected over the entire heart. Finally, cardiac localizers using the same single shot techniques are obtained to scout the standard cardiac planes: 2-chamber or vertical long axis (VLA), 4-chamber or horizontal long axis (HLA), 3-chamber, and short axis. These images can be collected fairly quickly, requiring no more than 5 min total for an experienced technologist.

#### Stress perfusion imaging

Stress first-pass myocardial perfusion imaging is the workhorse CMR method to detect myocardial ischemia. CMR-based perfusion imaging involves intravenous delivery of gadolinium-based contrast agent, with image acquisition designed to visualize the delivery (or lack thereof) of contrast from circulating blood to the myocardium. The effect of the contrast agent is to shorten the T1 value of surrounding tissues, which when used with a saturation recovery bSSFP or gradient echo sequence, leads to signal enhancement. Areas of the myocardium with reduced perfusion will appear hypointense due to the lower T1 in these areas compared with myocardial tissue with preserved perfusion. Data acquisition should be at each heartbeat using an acquisition window that is ideally 125 ms or shorter (but no longer than 165 ms), a slice thickness of 8–10 mm, and less than 3 mm in-plane spatial resolution.

Perfusion CMR is typically acquired in 3 short axis planes (base, mid and apical), with the option of adding a long axis plane as heart rate allows. The most reliable and rapid method to identify these planes is the ‘3 of 5’ method, where a stack of 5 slices is used for planning the stack position with the top and bottom slice positioned at the mitral valve plane and the apex of systolic scout images. The outer slices are then removed and only the inner 3 used for imaging. After planning, a test perfusion acquisition is often run without contrast or with a trivial amount of contrast and saline to insure adequacy of planes and intravenous access. The stress agent is then administered, either as a bolus (regadenoson, dipyridamole) or as a gradual infusion (adenosine). Once hyperemia has been achieved, the contrast agent is injected intravenously using a power injector at a rate of 3–7 mL/s; the dose of the contrast agent depends on the agent used and the size of the patient. A 30 mL saline chasing bolus should follow the contrast agent. The patient should be instructed to hold their breath as the contrast agent reaches the LV cavity to avoid motion artifacts or misalignment of the images which show myocardial enhancement. If motion correction techniques are available, these can be deployed with breath-holding to improve image quality, or to enable a free-breathing perfusion scan. Imaging should continue while the contrast agent passes through the LV myocardium (approximately 1 min following contrast injection). The total time for first pass perfusion imaging (patient preparation and recovery time) is around 5 min.

Often, perfusion imaging is repeated at rest after a delay of 10–15 min. With experience and consistency in perfusion image quality, rest perfusion imaging can be omitted to shorten scan times. A physician or nurse should be present during the stress scan to ensure patient safety during pharmacological stress, and can review the acquired images to determine if rest (or repeat stress) imaging is required.

#### Cine imaging

Cine images are typically acquired as a series of 2D images in rapid succession, played as movies to show the heart in motion. Cines imaging can be used for a variety of purposes, including assessing valve function, global and segmental contractility, wall motion abnormalities, and calculation of LVEF and RVEF. These images are typically captured with a temporal resolution high enough to enable evaluation of wall motion (< 45 ms/image) and high in-plane spatial resolution (~ 1.5 mm). Because the CMR raw (k-space) data must be collected over several heartbeats in a segmented fashion, these images are usually collected during a breath-hold to reduce artifacts due to patient respiration. Increasingly available free-breathing methods are relatively impervious to such artifacts.

Cine images are typically collected in several different orientations for complete assessment of regional and global function. A single slice each in VLA, HLA, and 3-chamber planes should be acquired at a minimum along with a stack of short axis cine acquisitions that cover both ventricles. This stack usually consists of 8–14 2D slices from base to apex with a slice thickness of 6–10 mm and a 0–4 mm gap totaling 10 mm per slice. For efficiency of CMR exam time, the short axis cine images are collected post-contrast to minimize non-scanning time prior to LGE imaging.

Cine acquisitions require scan times of 5–15 s for each 2D imaging slice location and are usually performed during a breath-hold. Collecting these images at end-expiration has been shown to provide more consistent positioning between slices, though ease of understanding and performing end-inspiration breathhold varies across patients [23]. Assuming that one cine series can be collected every 30 s (scan time plus recovery between breath-holds), and approximately 16 cine data sets must be collected, this set of scans requires around 8 min, making cines one of the more time-consuming portions of the CMR exam. Breath-hold cine scan time should be shortened by routinely reducing the field-of-view in the phase encoding direction, reducing the resolution in the phase encoding direction, increasing the read-out bandwidth, applying parallel imaging, and using partial Fourier techniques. In patients who have difficulty with breath-holding, cine imaging can be performed with (1) free breathing with multiple (i.e., 3–4) averages or (2) real-time acquisitions to mitigate respiration artifacts. As a reminder, adequate pre-scan effort in confirming the presence of a high quality gating signal will reduce the need for repeating image acquisition. Arrhythmia rejection is a tool provided by many vendors based on a range of algorithms. These tools can be helpful in improving image quality but can also lead to prolonged acquisition times and should generally be switched off in rapid CMR protocols. Prospective triggering is another option but warrants scrutiny to insure adequate coverage of the entire cardiac cycle.

#### Late gadolinium enhancement (LGE) imaging

LGE is an imaging technique in which static images of the heart are collected several minutes after intravenous administration of a gadolinium-based contrast agent. LGE is used to visualize myocardial damage across a variety of etiologies and is central to assessing the clinical indications in Table 1. Contrast agent which enters the myocardial tissue will wash out rapidly if the tissue is healthy but remains longer or in higher quantity in diseased tissue states with expanded extracellular space, leading to signal hyperintensity with suitableT1-weighted techniques. Acquisition of LGE images over 10 min post-contrast injection affords sufficient time for contrast to exit healthy tissue [59]. If stress perfusion is not being done as part of a rapid CMR protocol, contrast agent may be administered at the start of the exam or even before the patient is placed on the scanner table to avoid having to wait for the contrast agent to equilibrate.

LGE images are collected using inversion recovery (IR) segmented 2D inversion recovery bSSFP or gradient echo sequences. The timing of the data collection with respect to the IR pulse is crucial in order to “null” signal from healthy myocardium. An inversion time (TI) scout sequence can be used to determine the optimal TI that maximizes contrast between healthy and damaged myocardium. Newer scanners usually have phase-sensitive inversion-recovery (PSIR) sequences that are more robust to TI selection.

Once the optimal TI time has been determined, LGE images should be acquired in the same imaging planes as cine acquisitions. The in-plane spatial resolution and slice thickness should be similar to that used for cine imaging. Typically, only a single cardiac phase in end-diastole is collected for LGE images; thus, the acquisition window in each heartbeat can be much longer than that in cine scans but should not exceed 200 ms (shorter in patients with tachycardia). Due to the IR pulse, data can be collected only every other heartbeat, and thus standard LGE sequences require a ~ 10 s breath-hold. Like the cine scans, a total of ~ 30 s per image should be budgeted for planning and executing these acquisitions, making the total data collection time for LGE imaging 5–10 min (depending on the number of slices collected). As with cine imaging, strategies to shorten breath-hold duration include reducing the field-of-view in the phase encoding direction, reducing the resolution in the phase encoding direction, increasing the read-out bandwidth, applying parallel imaging, and using partial Fourier techniques. 3D LGE methods where available offer a time-efficient alternative to conventional 2D multi-slice acquisitions.

### Putting it together: the standard 30’ CMR exam

*Non-stress:* Following patient set up on the scanner, localization images are acquired to facilitate subsequent cardiac plane prescriptions. If no additional non-contrast scans are needed, gadolinium-based contrast agent can be administered, ideally via an automated pump from the CMR scanner control room. This is followed by cine and LGE imaging.

*Stress* Standard localizers are followed by long axis imaging to derive the LV short axis plane. A dummy perfusion scan is then performed. If this is satisfactory, the stress medication is administered, appropriately coupled to intravenous gadolinium-based contrast. If rest perfusion or repeat stress perfusion is not required, additional gadolinium-based contrast agent may be given. This is followed by cine images and LGE.

### Add-ons to the standard 30’ CMR exam

Once the referring physician becomes more knowledgeable about the capabilities of CMR to answer several clinical questions in one efficient exam, it is not uncommon for the CMR provider to receive a request to answer more than one question. In some cases, the ‘add-on’ questions can be addressed within a 30’ scanner time slot, or the scan time may be extended depending on the number of add-ons. For instance, in patients with non-ischemic cardiomyopathy, T1- and T2-weighted images of the myocardium may be acquired, or quantitative T1 and T2 mapping can be performed to better characterize the myocardium, to more precisely diagnose specific types of cardiomyopathy, and to follow treatment response in certain situations [60]. Additional cine and LGE planes tailored to the right heart (e.g., RV outflow, RV inflow) may be warranted in patients with right heart-related clinical questions, while standard cines yield the accurate RV quantification that is needed assess for arrhythmogenic cardiomyopathy [61].

In individuals with valvular heart disease or a cardiac shunt, phase contrast imaging can be to help quantify the degree of stenosis, regurgitation, or shunting. Velocity-encoded cines through the aortic root and pulmonary trunk can be acquired in a few minutes, adding little time to the protocol [62]. Similarly, an CMR angiogram (CMRA) can be performed during injection of the contrast agent in patients with suspected vascular disease or congenital heart disease. The need for these additional sequences is best determined through review of the patient’s history and review of prior records and coordination prior to the scan. The need may also be identified at the time of scanning by an experienced technologist who may recognize pathologies such as right heart enlargement, dephasing of blood flow through the valves, or dilation of the great arteries on scout images. A focus on answering the clinical questions by the CMR team helps avoid unnecessary protocol bloat.

The referring clinicians seek ease in ordering CMR for their patients. It is unreasonable to ask clinicians to include pulse sequence details or have an in-depth understanding of CMR imaging methodology. Similarly, we do not expect practitioners who order an echocardiogram to specifically ask for subcostal views or color flow Doppler on transthoracic echo orders. Similarly, the standardized 30-min ‘CMR should be as easy to order as an echocardiogram to answer those clinical questions most effectively answered by CMR. Efficient access to CMR for patients and referring physicians benefits from dedicated slots on the CMR scanner schedule and—as CMR volume grows—dedicated CMR scanners and centers. Encouraging CMR utilization for common clinical questions without ensuring timely access to the test is a recipe for disappointment, whereas routine use of a shorter CMR exam effectively increases scanner capacity and patient access.

## Conclusions and future directions

A number of common clinical questions can be addressed with a basic CMR exam that can easily be performed within 30 min or less on most scanners in any clinical practice setting. Members of the CMR team, partnering with facility stakeholders and referring clinicians is needed to improve access to CMR, and to translate decades of innovation to favorable impact the care of patients with a broad range of known or suspected cardiovascular disorders. Moreover, this efficient examination should be less cumbersome for both patients and technologists and can improve workflow for interpreting physicians. Not covered in this work is the importance of training for physicians and technologists seeking to advance access to CMR for their patients. Expanded training centers across regions can further support such clinicians growing CMR at both academic and private facilities. While this document has emphasized very well-established techniques, a follow-up document will be forthcoming that details some of the more contemporary techniques such as real-time imaging for even more efficient CMR examinations.

## Data Availability

Not applicable.

## Abbreviations

bSSFP: Balanced steady state free precession
CAD: Coronary artery disease
CMR: Cardiovascular magnetic resonance
CMRA: Cardiovascular magnetic resonance angiography
CT: Computed tomography
ECG: Electrocardiogram
FFR: Fractional flow reserve
FSE: Fast spin echo
HFmEF: Heart failure with mid-level ejection fraction
HFpEF: Heart failure with preserved ejection fraction
HFrEF: Heart failure with reduced ejection fraction
HLA: Horizontal long axis
ICD: Implanted cardioverter defibrillator
IHD: Ischemic heart disease
IR: Inversion recovery
LGE: Late gadolinium enhancement
LV: Left ventricle/left ventricular
LVEF: Left ventricular ejection fraction
LVOT: Left ventricular outflow tract
MINOCA: Myocardial infarction with no obstructive coronary arteries
MR: Magnetic resonance
PET: Positron emission tomography
PSIR: Phase sensitive inversion recovery
RV: Right ventricle/right ventricular
RVEF: Right ventricular ejection fraction
SAR: Specific absorption rate
SARS-CoV-2: Severe acute respiratory syndrome coronavirus-2
SNR: Signal-to-noise ratio
SPECT: Single photon emission computed tomography
TI: Inversion time
VLA: Vertical long axis
